# Synchrotron radiation micro X-ray fluorescence spectroscopy of thin structures in bone samples: comparison of confocal and color X-ray camera setups

**DOI:** 10.1107/S1600577516017057

**Published:** 2017-01-01

**Authors:** M. Rauwolf, A. Turyanskaya, A. Roschger, J. Prost, R. Simon, O. Scharf, M. Radtke, T. Schoonjans, A. Guilherme Buzanich, K. Klaushofer, P. Wobrauschek, J. G. Hofstaetter, P. Roschger, C. Streli

**Affiliations:** a Atominstitut, TU Wien, Vienna, Austria; b Ludwig Boltzmann Institute of Osteology at the Hanusch Hospital of WGKK and AUVA Trauma Centre Meidling, Vienna, Austria; c Forschungszentrum Karlsruhe/KIT, Institute for Synchrotron Radiation, ANKA, Karlsruhe, Germany; d IFG Institute of Scientific Instruments GmbH, Berlin, Germany; e Department of Analytical Chemistry, Bundesanstalt fuer Materialforschung und -pruefung, Berlin, Germany; f Orthopaedic Hospital Vienna-Speising, Vienna, Austria

**Keywords:** SR-µXRF, color X-ray camera, confocal, human bone

## Abstract

In the quest for finding the ideal synchrotron-radiation-induced imaging method for the investigation of trace element distributions in human bone samples, experiments were performed using both a scanning confocal synchrotron radiation micro X-ray fluorescence (SR-µXRF) (FLUO beamline at ANKA) setup and a full-field color X-ray camera (BAMline at BESSY-II) setup. As zinc is a trace element of special interest in bone, the setups were optimized for its detection. The setups were compared with respect to count rate, required measurement time and spatial resolution. It was demonstrated that the ideal method depends on the element of interest. Although for Ca (a major constituent of the bone with a low energy of 3.69 keV for its *K*α XRF line) the color X-ray camera provided a higher resolution in the plane, for Zn (a trace element in bone) only the confocal SR-µXRF setup was able to sufficiently image the distribution.

## Abstract 2

To find the ideal synchrotron radiation induced imaging method for the investigation of trace element distributions in bone tissue, experiments with a scanning confocal micro X-ray fluorescence system and a full-field color X-ray camera setup were performed.

## 1. Introduction   

Synchrotron radiation micro X-ray fluorescence (SR-µXRF) has proven to be a forceful method to perform imaging of trace elements in various materials (Janssens *et al.*, 2010 ▸; West *et al.*, 2015 ▸). SR-µXRF imaging techniques exist in full-field and in scanning mode. Whereas confocal SR-µXRF (a scanning-mode technique) for measurements of trace elements distributions in bone and cartilage has been successfully used before (Roschger *et al.*, 2013 ▸, 2010 ▸; Pemmer *et al.*, 2013 ▸, 2011 ▸; Smolek *et al.*, 2012 ▸; Zoeger *et al.*, 2008 ▸), the question remained whether or not a full-field technique would decrease measurement time and improve resolution.

To answer this question and to test the use of both modes for the analysis of thin bone microstructures such as tidemarks (transition zone between different phases of cartilage) and cement lines (the boundaries of osteons, the fundamental functional units of compact bone), we measured the same bone samples with two different systems: a confocal SR-µXRF setup and a color X-ray camera (full-field mode) setup.

A high fraction of the total zinc (Zn) in the body is stored in bone tissue. Zn is the co-factor of various metalloenzymes which play a major role in the mineralization process on new bone formation sites as well as in bone resorption and remodeling (Seo *et al.*, 2010 ▸; Beattie & Avenell, 1992 ▸). Additionally, increased Zn levels stimulate bone formation (Hall *et al.*, 1999 ▸; Yamaguchi & Yamaguchi, 1986 ▸). All this makes the analysis of Zn distributions in bone very intriguing. Therefore, both of the setups were optimized to measure Zn.

## 2. Materials and methods   

### 2.1. Samples   

Three human bone samples (seven regions of interest in total) were studied. These samples were vertebral bone tissues taken as biopsies from patients with vertebral compression fractures. Informed consent of all patients was obtained to use the material for research purposes. The study was conducted according to the guidelines of the Helsinki Declaration and approved by the ethics committee of the Medical University of Vienna, Austria.

The bone samples were undecalcified, dehydrated and fixed in a gradient of ethanol concentrations (50 to 100%) and embedded in polymethylmethacrylate. The sample blocks were trimmed [using a low-speed diamond saw from Buehler Isomet (Lake Bluff, IL, USA)], ground using sandpaper with decreasing grit size and finally polished with a precision polishing device (PM5, Logitech Ltd, Glasgow, UK). A more detailed description of the sample preparation is given by Roschger *et al.* (1998 ▸, 2008 ▸).

### 2.2. Quantitative backscattered electron imaging (qBEI)   

**Figure 1:**
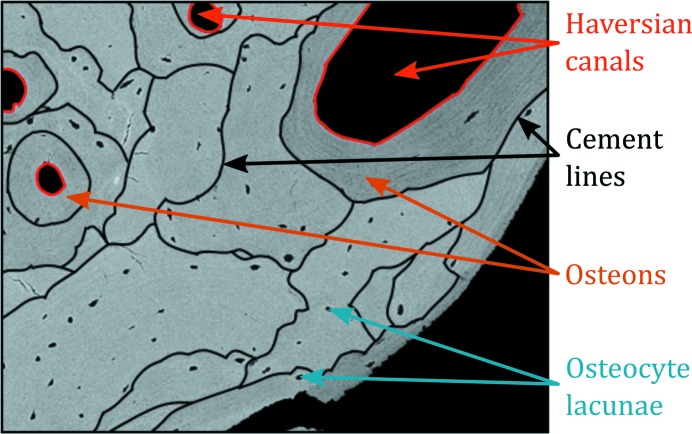
Cross section of cortical bone, a close-up from qBEI. Cement lines are marked in black.

The signal in qBEI is proportional to the average atomic number of the measured material. For bone, the hydroxy­apatite-like mineral phase dominates the signal, which allows one to determine the Ca content in each pixel of the imaged area. Bright areas in the qBEI image describe higher and dark areas lower Ca content (degree of tissue matrix mineralization). A digital scanning electron microscope (DSM 962, Zeiss, Oberkochen, Germany) equipped with four quadrant semiconductor backscattered electron detectors was operated at 20 keV beam energy and regions of interest were imaged with 200× nominal magnification (pixel resolution of 1 µm). A typical image from qBEI of cortical bone is shown in Fig. 1 ▸.

More information about qBEI can be found elsewhere (Roschger *et al.*, 1998 ▸, 2008 ▸, 2014 ▸; Fratzl-Zelman *et al.*, 2009 ▸).

### 2.3. SR-µXRF   

SR-µXRF is one of the most important variants of energy-dispersive X-ray fluorescence analysis (EDXRF). Measurements are performed on microscopically small areas of a larger sample. The technique is widely used for analytical tasks in different fields, such as geological sciences, art and archeology, environmental, biological and biomedical applications (Janssens *et al.*, 2010 ▸).

#### 2.3.1. Confocal SR-µXRF   

**Figure 2:**
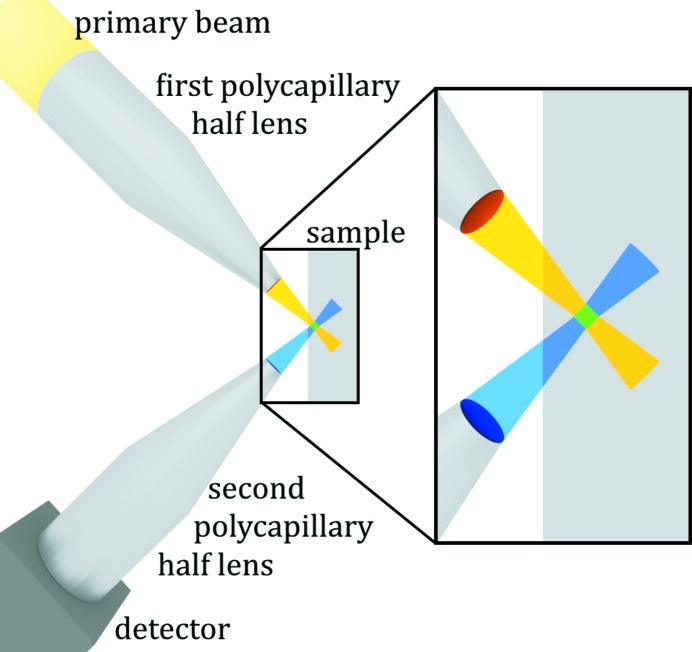
Schematic of the confocal SR-µXRF setup. The sample is scanned through a well defined detection volume (green square) created by the overlap of the focal spots of the polycapillary half-lenses. The detection volume of the setup at ANKA was 17 µm × 12 µm × 19  (depth) µm for Au *L*α.

In a confocal setup two X-ray optics (often polycapillaries) are used to define the sample volume from which the fluorescence radiation is detected. The first X-ray optics system focuses the primary beam on the sample. The second X-ray optics (a polycapillary half-lens) is placed between the sample and the detector. The angle between the two optics is 90°. The overlap of the focal spots of the two optics defines a volume from which the fluorescence radiation is seen by the detector. The sample can be moved through this detection volume. A schematic sketch of a confocal µXRF setup is shown in Fig. 2 ▸.

The confocal geometry offers improved measurement conditions as the measurement volume is now exactly defined by the overlapping focal spots of the two optics. The problem of different information depths for different elements (unwanted contributions from deeper layers) is minimized. Measurements in different layers of the sample are possible and can provide three-dimensional information.

Confocal SR-µXRF measurements were performed at the FLUO beamline at ANKA (KIT, Karlsruhe, Germany). The setup consisted of a W/Si double-multilayer monochromator and two polycapillary half lenses. The samples were excited at 17 keV and the fluorescence radiation was recorded with a 50 mm × 50 mm Vortex detector with an energy resolution (FWHM) of 160 eV for Mn *K*α. The detection volume was determined to be 17 µm × 12 µm (×19 µm depth) for Au *L*α (9.711 keV) by scanning 0.1 µm-thick gold (Au) microstructures. As the size of the detection volume is still somewhat energy dependent, slightly smaller step sizes (15 µm × 10 µm) were chosen for scanning the samples.

#### 2.3.2. Full-field SR-µXRF   

The color X-ray camera (CXC) (Scharf *et al.*, 2011 ▸; Ordavo *et al.*, 2011 ▸) installed at the BAMline at BESSY-II [Helmholtz-Zentrum Berlin fuer Materialien und Energie (HZB), Germany] works in full-field mode and allows both the detection of the energy of the X-ray photons as well as their spatial resolution. The CXC consists of a pn-junction charged-coupled device (pnCCD) and a polycapillary optics. The spatial resolution of the CXC is defined by the pnCCD pixel size (48 µm) and the polycapillary optics used.

**Figure 3:**
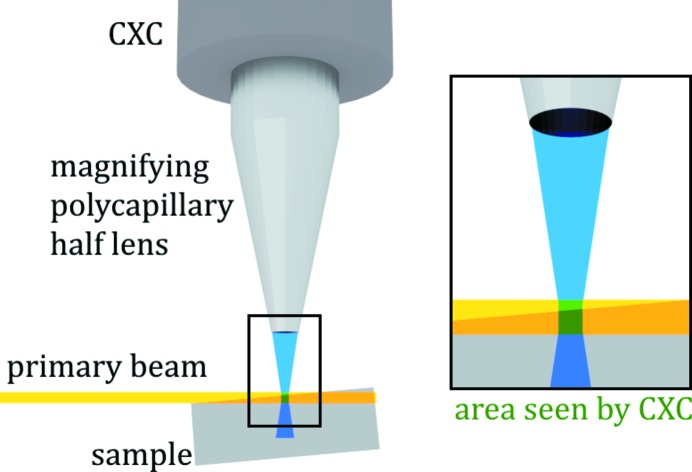
Schematic of the full-field CXC setup. The whole measured sample area (264 × 264 pixels) is excited at once (full-field mode).

For these measurements a conical-shaped magnifying polycapillary optics (magnification factor 1:8) was used. The spatial resolution was, therefore, about 7 µm × 7 µm in a single pixel. The depth resolution depends on the X-ray energy of the detected element as well as on the angle between the collimated primary beam and the sample, which was varied between 2.5° and 5°. The samples were excited at 12.6 keV in order to maximize the production of Zn XRF. The energy resolution of the CXC can be given as 200 eV for Mn *K*α. A schematic of the setup is shown in Fig. 3 ▸.

As one can see from Fig. 3 ▸, depending on the angle between the primary X-ray beam and the sample, the CXC can see deeper into the sample downstream from the primary X-ray beam.

## 3. Results   

The raw spectral data were analyzed using the *AXIL* and *MICROXRF2* software packages in order to extract the net-line XRF intensities for each measurement point (confocal XRF) or pixel (CXC), and convert them into elemental maps (Vekemans *et al.*, 1994 ▸).

**Figure 4:**
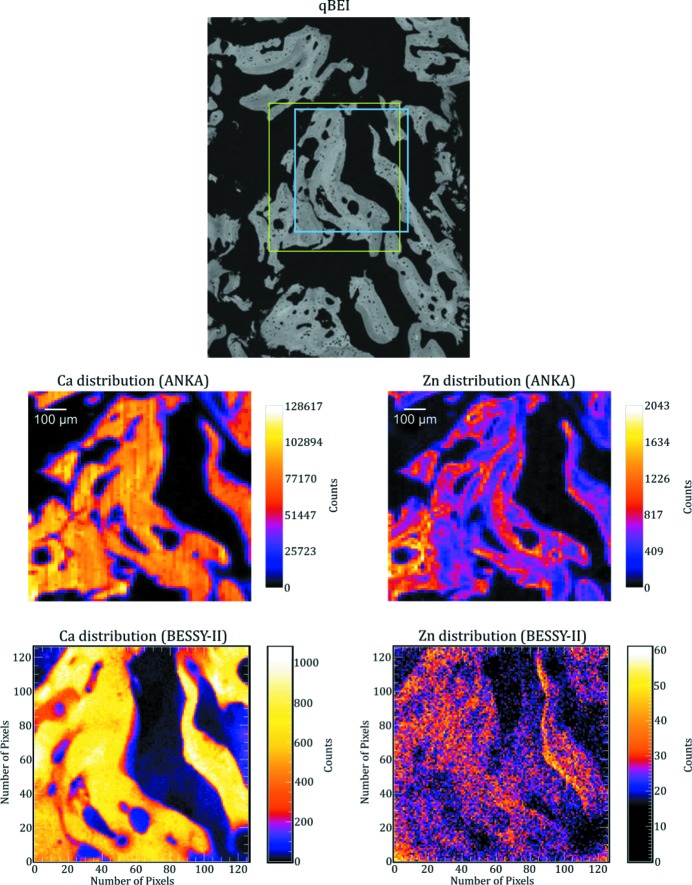
qBEI image as well as Ca and Zn distribution maps from approximately the same area measured at ANKA and BESSY-II. The exact area measured at ANKA is marked by a green rectangle in the qBEI image. Measurement time per voxel was 2 s (about 3.6 h for the complete map). The measurement performed at BESSY-II was made in the region marked in blue and took 5.5 h. The angle between the primary beam and the sample was about 2.5°.

As an example the Ca and Zn elemental maps as well as the corresponding qBEI images of two areas which were measured with both setups are shown in Figs. 4 ▸ and 5 ▸.

**Figure 5:**
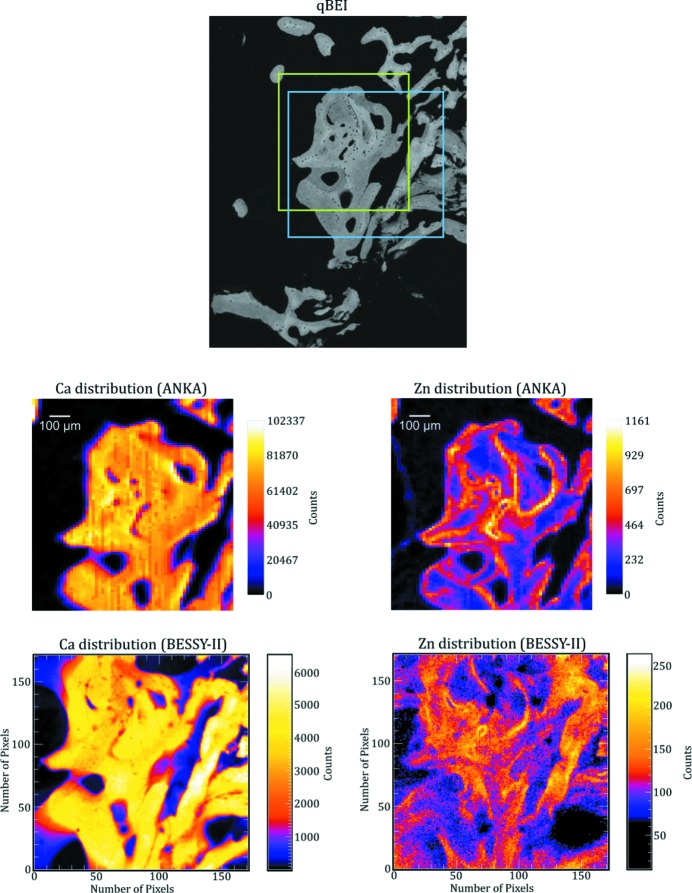
qBEI image as well as Ca and Zn distribution maps from approximately the same area measured at ANKA and BESSY-II. The exact area measured at ANKA is marked by a green rectangle in the qBEI image. Measurement time per voxel was 2 s (about 3.6 h for the complete map). The measurement performed at BESSY-II was made in the region marked in blue and took 18 h. The angle between the primary beam and the sample was about 5°.

Measurement time for the maps obtained in scanning mode (ANKA) was 2 s per pixel (about 3.6 h per map). The measurements in the full-field mode (BESSY-II) were performed: (*a*) with an angle of about 2.5° between the sample and the primary beam and 5.5 h measurement time (Fig. 4 ▸) and (*b*) with an angle of about 5° between the sample and the primary beam and a 18 h measurement time (Fig. 5 ▸).

As one can see from Figs. 4 ▸ and 5 ▸, we were able to image the Ca distribution with both setups quite satisfactorily. By comparing the Ca maps with the corresponding qBEI data (*e.g.* by observing the small holes in the bone in Fig. 4 ▸), one can easily see the higher resolution in the plane in the BESSY-II maps.

By observing bigger hole structures in the qBEI images and comparing them with the Ca maps one can see that, especially on the right-hand side of the BESSY-II maps, some structure from deeper layers is shown which is neither visible in the qBEI images nor in the ANKA maps. This leads to the conclusion that the depth resolution of the BESSY-II maps is worse compared with the ANKA maps. Additionally, it should be mentioned that the depth resolution of our measurements at BESSY-II is dependent on the angle between the sample and the primary beam and will be slightly different in different parts of the elemental maps (see Fig. 3 ▸).

Although the Ca distribution is clearly imaged in the BESSY-II maps the same does not hold for the Zn distribution. For the maps measured for 5.5 h with a 2.5° angle between the sample and the primary beam, the intensity was too low (and, therefore, the signal was too noisy) to reliably recognize the bone structure in the Zn distribution (see Fig. 4 ▸). For this reason, we measured the next sample for a longer time (18 h) and with a larger angle (about 5°). Although this improved the intensity by approximately a factor of five, the Zn distribution imaged in Fig. 5 ▸ shows a pattern that is not consistent with the qBEI image and the Ca distribution. The reason for this is the higher energy of the Zn *K*α line (8.6 keV) compared with the Ca *K*α line (3.69 keV), which results in a Zn signal with a worse depth resolution than the Ca signal. As the qBEI images have a depth resolution of 1 µm, the low depth resolution of the BESSY-II maps also makes it harder to correlate the elemental maps with the bone structure seen in the qBEI images.

## 4. Conclusion   

Although the CXC setup with the better resolution in the plane would be great to image the Ca distribution (one of the major elements in bone) in the samples, the count rates of Zn (trace element) were too low to take advantage of this. Enlarging the angle between the primary beam and the sample to obtain higher Zn count rates worsens the depth resolution and makes it harder to compare the elemental maps with the qBEI images from the surface. The confocal SR-µXRF setup at ANKA allowed us to image both Ca and Zn sufficiently, which made it possible to see a higher Zn accumulation in the cement line regions. However, as the cement line is only a few nanometers thin, the resolution is too low to investigate possible substructures. Furthermore, it should be mentioned that the ANKA synchrotron is no longer available for peer-reviewed user operation to external users. To investigate narrow bone structures and potential substructures further, a combination of SR-µXRF and transmission X-ray microscopy with thin samples could be fruitful. The use of thin samples could also solve the problem of restricted depth resolution for the CXC setup. Additionally, the CXC setup would benefit from optics with a higher efficiency to obtain results comparable with the confocal setup.
